# Masks, Gloves, Exports Licences and Composite Procedures: Implementing Regulation 2020/402 and the Limelight of Accountability

**DOI:** 10.1017/err.2020.28

**Published:** 2020-04-07

**Authors:** Luis ARROYO JIMÉNEZ, Mariolina ELIANTONIO

**Affiliations:** * Professor of Administrative Law, Jean Monnet Chair of European Administrative Law, University of Castilla-La Mancha, Ciudad Real, Spain; email: luis.arroyo@uclm.es.; ** Professor of European and Comparative Administrative Law and Procedure, Maastricht University, Maastricht, The Netherlands; email: m.eliantonio@maastrichtuniversity.nl.

## I. Introduction

The European Union (EU) response to the health emergency we are currently confronting has led to very different measures. Here, we will focus on one of them: Commission Implementing Regulation 2020/402, which imposes restrictions on exports of personal protective equipment with effect from 15 March 2020, for a period of six weeks, ending on 26 April 2020.^1^ Annex I of the Regulation defines personal protective equipment, by reference to Combined Nomenclature (CN) codes, as covering protective spectacles and visors, face shields, mouth–nose protection equipment, protective garments and gloves. In view of the current health emergency, personal protective equipment is defined as an essential product necessary to further prevent the spread of the COVID-19 disease and safeguarding the health of medical staff treating infected patients.^2^

Implementing Regulation 2020/402 reacts to a truly perfect storm. On the one hand, since internal demand for personal protective equipment has significantly increased in the last weeks, many Member States are experiencing shortages.^3^ On the other hand, some third countries that are also traditional suppliers to the Union market have decided to restrict exports, thus increasing scarcity in the internal market.^4^ Finally, production of personal protective equipment within the EU is currently concentrated in a limited number of Member States – the Czech Republic, France, Germany and Poland^5^ – and this creates the risk of national measures imposing restrictions on imports, exports or goods in transit justified on grounds of the protection of health.^6^

In view of the pressure that these three factors exert on the Union market, the Commission has decided to seal its external borders, in order to prevent personal protective equipment from being exported to other parts of the world on a general basis. In particular, Implementing Regulation 2020/402 requires an authorisation for the export outside the Union of personal protective equipment, whether or not it is originating in the Union.^7^

This measure is taken within the framework of the Regulation (EU) 2015/479, on common rules for exports,^8^ and it raises interesting questions from a trade law perspective.^9^ However, we will focus in this contribution on the administrative procedure for export authorisation, which may encompass the intervention of authorities from various Member States. While the authorities competent to grant the license are those where the applicant is established, if the protective equipment is located in one or more Member States other than the one where the application for export authorisation has been made, the opinion of the consulted Member State(s) is binding on the competent authorities.^10^ While this system serves the purposes of efficiency and speedy action required by the current emergency situation, it does raise a number of questions from the perspective of the accountability of the regulatory system set therein, particularly with reference to the availability of sufficient judicial control guarantees.

This contribution proceeds as follows. First, we place the administrative procedure sketched by Implementing Regulation 2020/402 in the context of the doctrinal discussion on the types of composite procedures. Subsequently, we present the judicial review concerns that can be raised with respect to this particular type of composite procedure, exploring some possible ways to address them in this particular case in view of how the Court of Justice has dealt in the past with somewhat similar cooperation arrangements. We then reach a conclusion on how to fill the gaps in the judicial control of the composite procedure for export authorisation, advancing two possible analogies with the case law of the Court of Justice.

## II. A horizontal composite procedure for export authorisation

Virtually all fields of EU law have witnessed the increasing use of regulatory structures that have come to be known as “composite procedures”. These can be defined as decision-making processes involving multiple jurisdictions participating at different moments and with different intensities.^11^ Several of them fall under the category of so-called “vertical” composite procedures, in the sense that the preparatory measures and the final decision in the decision-making process are taken by both national and European authorities. In turn, in “horizontal” composite procedures both the preparatory measures and the final decision are taken by national authorities,^12^ and the European authorities play no formal part in the decision-making process leading to the final decision. Implementing Regulation 2020/402 designs a composite authorisation procedure that belongs to this second category.

The administrative procedure formally begins with an application made by the exporter before the national authorities of the Member State where the exporter is established.^13^ If the personal protective equipment designated in the application is located in the territory of the Member State of the applicant, the procedure remains purely internal. In turn, if the equipment is located in one or more Member States other than the one where the export authorisation has been requested, the procedure will encompass a consultation with the administrative authorities of these other Member States.^14^ It is in these cases that the administrative decision-making process turns into a horizontal composite procedure. This is reflected in the following three features.

First of all, the fact that the products are located in other Member States is important in the very first stage of the procedure, since it is the duty of the party to indicate this circumstance in the application.^15^ Next, the competent authorities of the Member State to which the application for export authorisation has been made shall immediately consult the competent authorities of the other Member State or States in question. They must also provide them with all relevant information regarding the application.^16^ Finally, the Member State(s) consulted shall make known within 10 working days any objections they may have to the granting of such an authorisation. Crucially, the “objections” expressed by the consulted authorities “shall bind the Member State in which the application has been made”.^17^ This implies that consulted authorities enjoy a power to veto the granting of the license, which can be expressed through a preparatory opinion.

## III. In search of effective judicial protection

Composite procedures have not only attracted considerable scholarly attention in recent years,^18^ but also come increasingly often to the attention of the Court of Justice with the aim of clarifying, in particular, the judicial implications of this system of administrative governance.^19^ Indeed, whereas the system of decision-making for the implementation of EU law is increasingly composite in nature, the system of judicial protection has remained in principle anchored to a model based on domestic jurisdiction, whereby the court competent to review a certain administrative act or action is the court belonging to the legal system in which the act or action is imputable, regardless of whether it is part of a larger multi-jurisdictional decision-making process.^20^ The mismatch between the high integration of decision-making processes and the strict separation of control mechanisms may lead to various accountability gaps. From a judicial review perspective, these gaps have a specific constitutional dimension, since they reduce the effectiveness of judicial protection.^21^

In the context of the administrative procedure regulated by Implementing Regulation 2020/402, these gaps arise as a consequence of the fact that the binding opinion and the final decision are issued by authorities belonging to different Member States. This could lead to a lack of judicial protection if the application is rejected – or granted under conditions – by the competent authorities following an objection expressed by the consulted authorities.

On the one hand, private parties adversely affected by the final administrative decision should challenge it before the domestic courts of the Member State where the applicant is established. However, if the binding report issued by the consulted authorities of the other Member States vetoed the export, the competent authorities would have had no other option than abiding by it. At the same time, under national law, domestic courts reviewing the adverse final decision would have, in principle, no jurisdiction to review the preparatory measure taken by other national authorities.

On the other hand, trying to access the courts of the Member State where the products are located may provide no remedy, since the opposition to the export is expressed through a preparatory act that is part of a procedure that will be concluded by another administrative authority. Therefore, the preparatory measure is not intended to produce direct effects vis-à-vis the applicant. Indeed, in both EU administrative law and in the domestic administrative legal orders, preparatory measures – even if they are binding – are only rarely – if ever – open to direct judicial review.^22^ Therefore, a direct challenge by the applicant to the opinion issued by the consulted authorities before their national courts will hardly be admitted under national law.

The Court of Justice has not dealt specifically with gaps of judicial review in this kind of horizontal composite procedures. Nevertheless, it has developed two doctrines that might be explored in order to tailor a specific solution for them. Following the first path would lead to entrusting national courts of the Member State whose administrative authorities have taken the preparatory act with the responsibility of filling the gap. The second path would in turn confer this responsibility upon the courts of the Member State where the authorisation has been requested.

In *Borelli*, on the one hand, the Court dealt with a composite vertical procedure, the final decision of which was in the hands of the Commission, but where the national authority issued a binding preparatory measure. The national act could not be disregarded by the European authority^23^ and was not amenable to domestic judicial review under Italian procedural law. The Court then established three rules: first, that it had no jurisdiction to rule on the lawfulness of a measure adopted by a national authority;^24^ second, that any flaws of the national preparatory measure could not affect the validity of the decision taken by the European authority;^25^ and third – and crucially for our purposes – that national courts “must rule on the lawfulness of the national preparatory measure at issue on the same terms on which they review any definitive measure adopted by the same national authority which is capable of adversely affecting third parties, regardless of what domestic procedural law might establish in this respect”.^26^ This last rule was grounded on “the requirement of judicial control of any decision of a national authority reflects a general principle of Community law stemming from the constitutional traditions common to the Member States and has been enshrined in Articles 6 and 13 of the European Convention for the Protection of Human Rights and Fundamental Freedoms”.^27^

The regulatory setup created by Implementing Regulation 2020/402 is different from the one at stake in *Borelli* in that the structure of the procedure is not vertical, but horizontal. Nevertheless, the two procedures display at least two similarities: first, as in *Borelli*, the final decision-maker is bound by the result of the preparatory measure. Second, in both cases, restrictions imposed by internal procedural legislation regarding the reviewability of administrative decisions impede domestic judicial control of the national preparatory measure.

These features might allow an analogical application of the *Borelli* ruling to the binding opinion provided for by Implementing Regulation 2020/402. Domestic courts of the Member State where the goods are located should, on this basis, review the legality of the administrative opinion expressing objections or imposing conditions as if it was a final administrative decision capable of adversely affecting third parties. Therefore, they should “regard an action brought for that purpose as admissible even if the domestic rules of procedure do not provide for this in such a case”.^28^

On the other hand, the Court dealt in *Berlioz* with a horizontal cooperation arrangement between the tax administrations of two Member States.^29^ French authorities had sent the Luxembourg tax administration a request for information concerning Berlioz pursuant, in particular, to Directive 2011/16.^30^ Following that request, the Luxembourg authority took a decision requiring Berlioz to provide certain information. Since Berlioz did not fully comply with the request, the Luxembourg tax administration imposed an administrative fine on account of its refusal to provide that information. According to national law, Berlioz could challenge the administrative fine (which was a measure taken by the Luxembourg authorities), but not the request for exchange of information, nor the decision requiring the requested information to be provided (which were measures issued by a foreign administrative authority). The Court of Justice established that the right to effective judicial protection enshrined in Article 47 of the Charter, and specifically the right to access to an independent and impartial tribunal, requires that “a decision of an administrative authority … must be subject to subsequent control by a judicial body that must, in particular, have jurisdiction to consider all the relevant issues”.^31^ Consequently, Luxemburg courts “hearing an action against the pecuniary administrative penalty imposed on [Berlioz] for failure to comply with an information order must be able to examine the legality” of the latter.^32^ Moreover, the right to effective judicial protection also demands that, when reviewing the legality of the information order, the Court “carry out the review of the legality of the request for information”,^33^ with the limitations that also apply to the requested administrative authority.^34^

The regulatory arrangement designed by Implementing Regulation 2020/402 differs from the *Berlioz* case, since in the latter the Court was not dealing with a decision-making process, but with an enforcement measure as a form of administrative cooperation. However, there are also certain similarities that relate to relevant features in terms of whether and how judicial gaps must be filled up. In both cases, under domestic law, national courts have, in principle, no jurisdiction to review the legality of foreign administrative measures. Moreover, challenging these measures before the courts of the Member State whose administrative authorities issued them might well be precluded under national law because of their preparatory nature. In both cases, the setup leads to a severe reduction of the effectiveness of judicial protection, since the legality of the relevant preparatory measure might not be reviewed by any national jurisdiction. As the Court made clear in *Berlioz*, these situations are prohibited by Article 47 of the Charter.

It would therefore seem plausible to analogically apply the *Berlioz* solution to the judicial review of other horizontal composite procedures, such as the one established by Implementing Regulation 2020/402. Accordingly, national courts of the Member State whose authorities have rejected – or conditioned – the authorisation must be able to “carry out the review of the legality” of the preparatory act in which foreign authorities expressed their objections to authorise the export – or required the imposition of certain conditions – despite it being issued by the administrative authorities of another Member State.

## IV. Conclusion

Implementing Regulation 2020/402 creates a horizontal composite procedure for export authorisations. The authorities competent to take the final decision in the procedure are those of the Member State where the applicant is established. However, if the equipment is located in the territory of (an)other Member State(s), the administrative authorities of the latter will have to issue a preparatory – binding – opinion. Judicial review of this binding opinion might be precluded by national law, and this gap might entail the violation of the fundamental right to an effective remedy enshrined in Article 47 of the Charter.

This is but another expression of the mismatch between how power is exercised in the European administrative union – procedural integration – and how accountability unfolds in both the political and the judicial arenas – formal separation. We have advanced in this contribution two possible avenues to overcome this situation.

On the one hand, the gap could be filled up by analogically applying the *Borelli* doctrine. This would lead to affirming the duty of the domestic courts of the Member State where products are located to review the legality of the binding opinion expressing objections or imposing conditions as if it was a final administrative decision capable of adversely affecting third parties.

On the other hand, the gap could also be filled up through an analogical reading of the *Berlioz* ruling. This second path would lead to recognising that the courts of the Member State taking the final decision on the procedure would have jurisdiction to examine the legality of the preparatory opinion issued by the consulted authorities. However, this scenario raises important – and unanswered – questions: is the review of the court limited to the violation of EU law, or does it extend to possible violations of national law? What are the consequences of a national court ruling on the unlawfulness of a foreign administrative act? To what extent should the principle of *res judicata* apply in such situations?

Both *Borelli* and *Berlioz* are decided in respect to situations that are somewhat different from the cooperation arrangement established by Implementing Regulation 2020/402. However, both scenarios display similarities with the one contained in Implementing Regulation 2020/402, and they provide possible solutions as to how the gap of judicial review could be filled. While the final word unequivocally rests with the Court of Justice, it should be noted that various arguments could be brought in favour of a *Berlioz*-like solution over a *Borelli*-like one. First of all, admitting that the court competent for the review of the final measure in the decision-making process would be able to also review a foreign preparatory step would seem more “protective” for the individual who would – unlike the *Borelli* scenario – initiate only one judicial review procedure, covering the entirety of the process. Secondly, since the binding opinion may well not even be notified to the applicant, the *Berlioz* solution would guarantee a more effective judicial protection. Finally, the system of “integrated judicial review” proposed by *Berlioz* seems more in line with the increasingly integrated system of decision-making in EU administrative governance.

## Footnotes

1 Commission Implementing Regulation (EU) 2020/402 of 14 March 2020 making the exportation of certain products subject to the production of an export authorization C/2020/1751 (OJ 2020 L 771 p 1).

2 ibid, Recital 2.

3 ibid, Recital 5.

4 ibid, Recital 6.

5 ibid, Recital 5.

6 Treaty on the Functioning of the European Union, Art 30.

7 Implementing Regulation (EU) 2020/402, supra, note 1, Art 1.

8 Regulation (EU) 2015/479 of the European Parliament and of the Council of 11 March 2015 on common rules for exports (OJ 2015 L 83 p 34).

9 See, eg, I van Damme, “European Union Imposes Export Restrictions on Personal Protective Equipment”, *EU Law Live*, 17 March 2020 <https://eulawlive.com/analysis-european-union-imposes-export-restrictions-on-personal-protective-equipment-by-isabelle-van-damme>; and P Oliver, “COVID-19 and the Free Movement of Goods: Which Prevails?”, *EU Law Live*, 19 March 2020 <https://eulawlive.com/analysis-covid-19-and-the-free-movement-of-goods-which-prevails-by-peter-oliver>.

10 The structure of the decision-making process is similar to that of the procedure for the authorisation of certain individual exports of dual-use items provided for in Art 11 of Council Regulation (EC) No 428/2009 of 5 May 2009, setting up a Community regime for the control of exports, transfer, brokering and transit of dual-use items (OJ 2009 L 134 p 1).

11 For this definition, see M Eliantonio, “Judicial Review in an Integrated Administration: The Case of ‘Composite Procedures’” (2014) 7 Review of European Administrative Law 65.

12 HCH Hofmann, GC Rowe and AH Türk, Administrative Law and Policy of the European Union (Oxford, Oxford University Press 2011) p 408.

13 Implementing Regulation 2020/402, supra, note 1, Art 1(1).

14 ibid, Art 2(1).

15 ibid, Art 2(1).

16 ibid, Art 2(1).

17 ibid, Art 2(1).

18 M Eliantonio, supra, note 11; F Brito Bastos, “The *Borelli* Doctrine Revisited: Three Issues of Coherence in a Landmark Ruling for EU Administrative Justice” (2015) 8 Review of European Administrative Law 269; F Brito Bastos, “Derivative Illegality in European Composite Administrative Procedures” (2018) 55 Common Market Law Review 101; S Alonso de León, *Composite Administrative Procedures in the European Union* (Ciudad Real, Iustel/UCLM 2017) pp 251–318; J Fernández Gaztea, “A Jurisdiction of Jurisdictions” (2019) 12 Review of European Administrative Law 9. With regard to composite procedures involving agencies, see M Scholten and M Luchtman (eds), Law Enforcement by EU Authorities: Implications for Political and Judicial Accountability (Cheltenham, Edward Elgar 2017). With regard to composite procedures involving data-sharing activities, see the special issue 20(1) of European Public Law (2014). In relation to pharmaceutical authorisations, see S Röttger-Wirtz and M Eliantonio, “From Integration to Exclusion: EU Composite Administration and Gaps in Judicial Accountability in the Authorisation of Pharmaceuticals” (2010) 10 European Journal of Risk Regulation 393.

19 See, eg, case C-562/12 *Liivimaa Lihaveis MTÜ* [2014] EU:C:2014:2229.

20 HCH Hofmann and M Tidghi, “Rights and Remedies in Implementation of EU Policies by Multi-Jurisdictional Networks” (2014) 20(1) European Public Law 154.

21 Charter of Fundamental Rights of the EU, Art 47. See G Sydow, Verwaltungskooperation in der Europäischen Union (Tübingen, Mohr Siebeck, 2004) pp 277–296; L de Lucia, “Administrative Pluralism, Horizontal Cooperation and Transnational Administrative Acts” (2012) 5 Review of European Administrative Law 17.

22 For the Italian, German and English legal systems, see M Eliantonio, Europeanisation of Administrative Justice? The Influence of the ECJ’s Case Law in Italy, Germany and England (Zutphen, Europa Law Publishing, 2008) Ch 1. This problem has again been highlighted recently by AG Bobek in the context of a report issued by a Member State and addressed to another Member State that constitutes a preparatory step in a marketing authorisation procedure: “[I]n a number of Member States, it is quite likely that that report may be classified as a preparatory act and thus not amenable to judicial review”. Opinion of AG Bobek in Case C-557/16, *Astellas Pharma GmbH*, ECLI:EU:C:2017:957, para 90.

23 Regulation (EEC) No 355/77 of the Council, of 15 February 1977, on common measures to improve the conditions under which agricultural products are processed and marketed (OJ 1977 L 51, p 1).

24 Case C-97/91, *Oleificio Borelli v Commission* EU:C:1992:491, paras 9–11.

25 ibid, para 12.

26 ibid, para 13.

27 ibid, para 14.

28 ibid, para 13.

29 Case C-682/15 *Berlioz* [2017] ECLI:EU:C:2017:373.

30 Directive 2011/16/EU of the Council, of 15 February 2011, on administrative cooperation in the field of taxation and repealing Directive 77/799/EEC (OJ 2011 L 64, p 1).

31 Case C-682/15 *Berlioz*, supra, note 29, para 55.

32 ibid, para 56.

33 ibid, para 84.

34 ibid, para 85.

